# Single-stranded positive-sense RNA viruses generated in days using infectious subgenomic amplicons

**DOI:** 10.1099/vir.0.068023-0

**Published:** 2014-11

**Authors:** Fabien Aubry, Antoine Nougairède, Lauriane de Fabritus, Gilles Querat, Ernest A. Gould, Xavier de Lamballerie

**Affiliations:** Aix Marseille Université, IRD French Institute of Research for Development, EHESP French School of Public Health, EPV UMR_D 190 ‘Emergence des Pathologies Virales’, 13385 Marseille, France

## Abstract

Reverse genetics is a key methodology for producing genetically modified RNA viruses and deciphering cellular and viral biological properties, but methods based on the preparation of plasmid-based complete viral genomes are laborious and unpredictable. Here, both wild-type and genetically modified infectious RNA viruses were generated in days using the newly described ISA (infectious-subgenomic-amplicons) method. This new versatile and simple procedure may enhance our capacity to obtain infectious RNA viruses from PCR-amplified genetic material.

## Introduction

Development of molecular methods that enable production of infectious virus from DNA copies of their genomes has significantly improved our knowledge of RNA virus life cycles and pathogenesis, by permitting the development of ‘reverse genetics’, i.e. studies of the impact of specific mutations on the biological properties of viruses (Gorchakov *et al.*, 2012; Ruggli & Rice, 1999). However, current methodologies for construction of infectious cDNA clones are unpredictable and laborious processes frequently associated with undesirable mutations or unstable/toxic clones in bacteria (Ruggli & Rice, 1999). Various methodological improvements, such as the use of alternative hosts (Csörgo *et al.*, 2012; Polo *et al.*, 1997; Schoggins *et al.*, 2012), low-copy-number plasmids (Bredenbeek *et al.*, 2003; Gritsun & Gould, 1998), cosmid vectors (Zhang *et al.*, 2001), bacterial artificial chromosomes (Suzuki *et al.*, 2007), modified promoters (Mishin *et al.*, 2001) or modified viral genome sequences with reduced cryptic bacterial promoter activity (Pu *et al.*, 2011) have been proposed. More recently, a new method was described to propagate flavivirus infectious cDNA clones in bacteria by introducing tandem repeat sequences upstream of virus genome (Pu *et al.*, 2014). Bacterium-free approaches have also been developed, for example with tick-borne encephalitis virus (TBEV) by Gritsun & Gould (1995) and with West Nile virus (WNV) and dengue virus (DENV) by Edmonds *et al.* (2013) and Siridechadilok *et al.* (2013), respectively. Although they represented significant advances, these methods require substantial optimization for each virus studied and do not provide a unified methodological process.

In the current study, we describe a simple and versatile reverse genetics method designated ISA (infectious-subgenomic-amplicons) that facilitates the rescue of infectious RNA viruses from genomic DNA material without requiring cloning, propagation of cDNA into bacteria or *in vitro* RNA transcription. Our working hypothesis was that transfection of overlapping double-stranded DNA fragments, covering the entire genome of an RNA virus, into susceptible animal cells would spontaneously enable recombination and synthesis of a DNA copy of the complete viral genome. By including at the 5′ terminus of the first (5′) DNA fragment a promoter of DNA-dependent RNA polymerases, and at the 3′ terminus of the last (3′) DNA fragment a ribozyme sequence and a signal sequence for RNA poly-adenylation, we anticipated that this genomic DNA copy would be transcribed as a full-length RNA genome with authentic 5′ and 3′ termini that would be efficiently exported out of the nucleus (in the case of a virus replicating in the cytoplasmic compartment).

## Results and Discussion

**Fig. 1.**
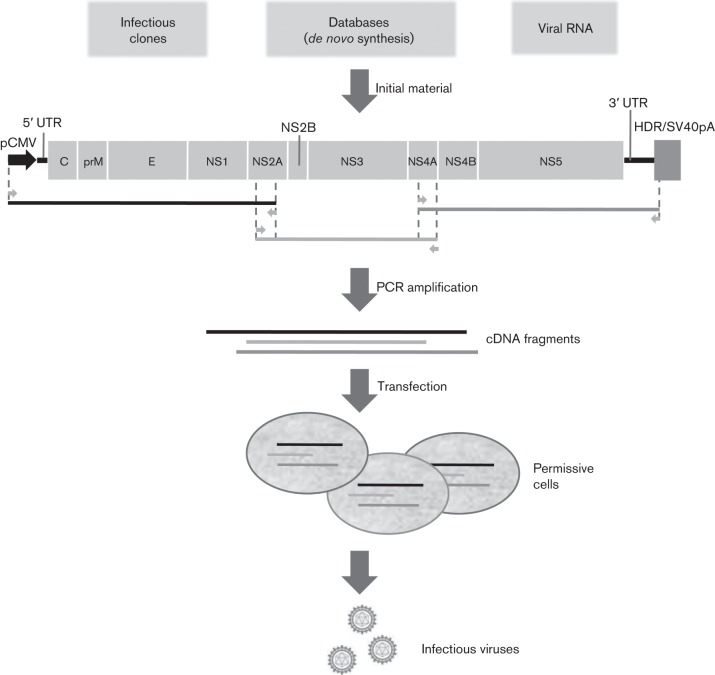
Universal strategy to rescue infectious single stranded positive RNA viruses. The entire viral genome, schematically represented in the figure (flaviviral genome), flanked respectively at the 5′ and 3′ untranslated regions (UTRs) by the human cytomegalovirus promoter (pCMV) and the hepatitis delta ribozyme followed by the simian virus 40 polyadenylation signal (HDR/SV40pA), was amplified by PCR in three overlapping cDNA fragments. Transfection of PCR products into permissive cells enabled the recovery of infectious viruses after 3 to 9 days. Horizontal grey arrows represent primers used to generate the three overlapping cDNA fragments.

We first tested this hypothesis with six flaviviruses (i.e. arthropod-borne enveloped viruses with a single-stranded RNA genome of positive polarity that replicate in the cytoplasm of infected cells) that represent major flaviviral evolutionary lineages. We used two Japanese encephalitis viruses (JEVs), genotype I (JEV I) and genotype III (JEV III), one genotype 2 WNV, one serotype-4 DENV, one wild-type (WT) strain of Yellow fever virus (YFV) and one Far-Eastern subtype TBEV (Table 1). Entire genomes were amplified by PCR in three DNA fragments of approximately 4 kb, each with 70–100 bp overlapping regions. The first and last fragments were flanked respectively at 5′ and 3′ by the human cytomegalovirus promoter (pCMV) and the hepatitis delta ribozyme followed by the simian virus 40 polyadenylation signal (HDR/SV40pA) (Fig. 1). PCR products were column-purified, and 1 µg of an equimolar mix of all fragments was transfected into human adrenal carcinoma (SW13) and/or baby hamster kidney (BHK)-21 cell lines, which, in our experience, ensure efficient recovery of flaviviral infectious genomes. Cell supernatant media from these infectious cultures were serially passaged four times using the same cell types, enabling the isolation of JEV I, JEV III, TBEV and WNV. For more demanding viruses, isolation could be achieved by passaging in additional permissive cells, such as DENV-4 in African green monkey kidney (VeroE6) cells or YFV in human embryonic kidney (HEK)-293 cells. Virus replication after four serial passages was demonstrated for each virus using a combination of the following criteria: (i) production of viral genomes in cell supernatant medium using real-time reverse transcriptase (RT)-PCR methods, (ii) production of infectious particles in cell supernatant medium using TCID_50_ assays, (iii) detection of cytopathic effect (CPE), (iv) detection of viral antigens by direct immunofluorescence assays and (v) complete viral genome sequencing using the next generation sequencing (NGS) method (Fig. S1 available in the online Supplementary Material, Table 1). Four passages were made for each virus produced using the ISA method to ensure the complete disappearance of the DNA used during the transfection.

**Table 1.** Characterization of the recovered viruses and strains in this study Origin of the initial material for production of the first (I), second (II) and third (III) fragments (DNS, *de novo* Synthesis; I.C., infectious clone; or viral RNA) used as the template, cell lines used for transfection and passage, relative quantification of the amount of viral RNA and infectious titres in cell supernatants at the fourth passage by real-time RT-PCR and TCID_50_ assay, presence or absence of cytopathic effect (CPE), direct immunofluorescence assay (dIFA) of viral antigens, ratio of number of non-synonymous substitutions per non-synonymous site (dN) to number of synonymous substitutions per synonymous site (dS) and substitutions per site are summarized. Characteristics of the mutations detected including nature, localization and frequency are detailed in Table S3.

Virus	Strain	Origin of template for subgenomic amplicons			Cell line		Real-time RT-PCR (AU)	Log_10_ TCID_50_ ml^−1^	CPE	dIFA	dN/dS (all mutations)	dN/dS (fixed mutations)	Substitutions per site after 4 passages (all mutations)	Substitutions per site after 4 passages (fixed mutations)
		I	II	III	transfection	passage								
JEV	JEV I	DNS	DNS	DNS	BHK-21	BHK-21	1.32E+08	5.8	Yes	na	3.273	na	1.27E-03	7.29E-04
SW13	SW13	1.52E+07	5.2	Yes	Positive	0.409	na	7.29E-04	9.11E-05
SW13*	SW13*	9.33E+06	2.8*	Yes	na	na	na	na	na
JEV III	I.C.	I.C.	I.C.	BHK-21	BHK-21	3.77E+07	6.1	Yes	na	1.286	1.143	1.54E-03	1.45E-03
SW13	SW13	4.04E+06	4.8	Yes	Positive	0.536	na	6.37E-04	–
Chimeric JEV I/JEV III	DNS	I.C.	I.C.	BHK-21	BHK-21	9.33E+07	6.7	Yes	na	0.404	1.571	1.36E-03	3.64E-04
SW13	SW13	1.00E+07	6.8	Yes	na	1.19	1.589	9.10E-04	7.28E-04
Chimeric JEV III/JEV I	I.C.	DNS	DNS	BHK-21	BHK-21	6.58E+07	6.6	Yes	na	0.268	0.268	2.73E-04	2.73E-04
SW13	SW13	3.06E+07	6.4	Yes	na	5.357	3.178	1.00E-03	6.38E-04
WNV	Ouganda	I.C.	I.C.	I.C.	BHK-21	BHK-21	5.73E+07	5.3	Yes	na	0.268	na	4.55E-04	2.73E-04
TBEV	Oshima 5.10	I.C.	I.C.	I.C.	BHK-21	BHK-21	3.28E+08	9.1	Yes	na	3.214	na	7.20E-04	9.00E-05
DENV-4	Dak HD 34 460	DNS	Viral RNA	DNS	SW13	VeroE6	6.59E+04	na	No	Positive	0.436	0.535	8.45E-04	5.63E-04
YFV	BOL 88/1999	DNS	Viral RNA	DNS	SW13	HEK	1.42E+05	5.2	Yes	na	0.818	0.818	4.63E-04	4.63E-04
CHIKV	OPY1	I.C.	I.C.	I.C.	HEK-293	HEK-293	2.01E+07	7	Yes	na	2.24	na	4.21E-04	–
CV-B3	2679	I.C.	I.C.	I.C.	SW13	BGM	4.64E+07	7.4	Yes	na	na	na	2.70E-04	–
CV-B3 †	2679†	Not obtained by PCR†			SW13†	BGM†	9.33E+07	7.4†	Yes	na	na	na	–	–

na, not available; AU, arbitrary unit.

* Results obtained by transfection of six overlapping fragments.

† Results obtained by directly transfecting the CV-B3 plasmid-bearing infectious clone.

The robustness, flexibility and versatility of the methods were further challenged as follows.

Firstly, we decreased the size and increased the number of overlapping fragments combined for transfection. This was exemplified in the case of JEV I, for which the ISA method generated infectious viruses, when using up to six overlapping amplicons of approximately 2 kb.

Secondly, we applied the ISA method to viruses with a single-stranded RNA genome of positive polarity that belong to different families: Chikungunya virus (CHIKV, an enveloped virus, family *Togaviridae*) and coxsackievirus B3 (CV-B3, a non-enveloped virus, family *Picornaviridae*). Again, infectious viruses could be isolated following transfection and four passages in HEK-293 cells (CHIKV) or Buffalo green monkey (BGM) cells (CV-B3) (Table 1). Furthermore, we used the CV-B3 obtained following transfection of a plasmid-bearing infectious genome as a control, and we obtained similar results in terms of infectivity and sequence data (Table 1).

Thirdly, we demonstrated the capability of the ISA method to generate genetically modified viruses in days. This was exemplified by the PCR-based correction of a frame-shift mutation (1915del) in fragment one of a defective JEV III infectious clone and the subsequent recovery of the corresponding virus (Supplementary Methods). We were also able to produce chimeric viruses by exchanging the first DNA fragment (encoding structural proteins) of genotype I and III JEVs. Despite 11 mismatches in the overlapping region of the first two fragments, transfection resulted in the production of inter-genotypic JEV I/JEV III and JEV III/JEV I chimeras (Table 1).

Analysis of complete genomic sequences established at the fourth passage, using NGS, showed that the genetic drift (rate of sequence change) was modest (ranging from 1.45×10^−3^ to 9.00×10^−5^ substitutions per site when considering fixed mutations) (Table 1). A majority of non-synonymous mutations, the presence of shared mutations amongst the different JEV strains (7/85, Table S1), and the non-random distribution of mutations at a frequency above 10 % along the genome, with both hot spots and highly conserved regions (Fig. S2), denoted adaptation to the cell culture conditions. The mutation rate varied according to the cells used for isolation, and as expected, was higher in viruses derived from low-passage strains than in those derived from culture-adapted strains.

In conclusion, the ISA method is a simple procedure with which to expedite production of infectious WT or genetically modified animal RNA viruses within days, with great control of the viral sequences and starting from a variety of initial sources including pre-existing infectious clones, viral RNA or *de novo* synthesized DNA genomic sequences. Unlike other bacterium-free approaches, the ISA method does not require any additional steps beside the PCR amplification needed to obtain the different cDNA fragments. The assembly of the construct is not produced *in vitro* by Gibson assembly or circular polymerase extension cloning before the transfection, but by *in cellulo* recombination, which greatly facilitates and shortens the method. Such an *in cellulo* recombination process was previously proposed in the case of plant viruses (Marillonnet *et al.*, 2004), but the *in planta* method proposed was quite different since (i) DNA fragments were provided in the form of plasmids; (ii) they were not transfected, but brought by agroinfiltration of *Nicotiana benthamiana* by a mix of *Agrobacterium* strains carrying provector plasmids; and (iii) a site-specific recombination was used that required the introduction of a specific sequence within the construct.

This novel technique has the potential to generate the design of large reverse genetics experiments for RNA viruses on a scale that could not previously have been considered (i.e. generating dozens or even hundreds of modified viruses at the same time). It also has the capacity, specifically, to modulate the characteristics of the viruses recovered from experimental procedures. Additionally, because DNA subgenomic fragments can conveniently be obtained by PCR, this method allows the conservation of the genetic diversity of viral populations (Edmonds *et al.*, 2013) when starting from viral RNA. Error-prone PCR may also be used to create artificial viral heterogeneity, e.g. for facilitating the selection of adapted viruses (Bacher *et al.*, 2002) under various experimental selection conditions, and conversely, high-fidelity polymerases and clonal amplification templates may be used to control the degree of clonality of the viruses produced. Finally, the ISA method has the potential to profoundly improve the safety and security of future exchanges of RNA viruses between scientific institutions by the separate shipment at room temperature of simple, non-infectious, DNA subgenomic fragments that could then be combined and transfected by the recipient institute, enabling rapid, simple and safe recovery of the infectious viral strain.

## Methods

### 

#### Cells, viruses, infectious clones and antibodies.

BHK-21 cells were grown at 37 °C with 5 % CO_2_ in a minimal essential medium (Life Technologies) with 7 % heat-inactivated foetal bovine serum (FBS; Life Technologies) and 1 % penicillin/streptomycin (PS; 5000 U ml^−1^ and 5000 µg ml^−1^; Life Technologies). HEK-293 cells and VeroE6 cells were grown at 37 °C with 5 % CO_2_ in the same medium as BHK-21 cells supplemented with 1 % non-essential amino acids (Life Technologies). SW13 cells were grown at 37 °C with 5 % CO_2_ in RPMI 1640 medium (Life Technologies) with 10 % FBS and 1 % PS.

JEV genotype I strain JEV_CNS769_Laos_2009 (KC196115) was isolated in June 2009 from the cerebrospinal fluid of a patient in Laos (Aubry *et al.*, 2013); YFV strain BOL 88/1999 (KF907504), isolated in 2009 from human serum, was kindly provided by the National Center of Tropical Diseases (CENETROP), Santa-Cruz, Bolivia; DENV-4 strain Dak HD 34 460 (KF907503), isolated from human serum, was kindly provided by Robert B. Tesh from the Center for Biodefence and Emerging Infectious Diseases–Sealy Center for Vaccine Development (University of Texas Medical Branch, Galveston, Texas, USA); the infectious clone of JEV genotype III derived from the strain rp9 (DQ648597) was kindly provided by Yi-Ling Lin from the Institute of Biomedical Sciences, Academia Sinica, Taipei, Taiwan; the infectious clone of WNV was derived from the strain Ouganda 1937 (M12294); the infectious clone of TBEV was derived from the strain Oshima 5.10 (AB062063); the infectious clone of CV-B3 was derived from the strain 2679 (KJ489414).

A JEV-specific immune serum (obtained after vaccination against JEV) and monoclonal DENV-specific antibodies (Henchal *et al.*, 1982) were used to perform direct immunofluorescence assays.

#### Preparation of cDNA fragments.

The complete genome flanked respectively at 5′ and 3′ by the human cytomegalovirus promoter (pCMV) (Note S1) and the hepatitis delta ribozyme followed by the simian virus 40 polyadenylation signal (HDR/SV40pA) (Note S2) was amplified by PCR in three overlapping DNA fragments of approximately 4.8 kb, 3.0 kb and 4.3 kb (4.8 kb, 2.9 kb and 5.2 kb for CHIKV, 4.8 kb, 4.1 kb and 3.4 kb for TBEV and 2.9 kb, 2.8 kb and 2.7 kb for CV-B3) (Table S4). For WNV, TBEV, JEV III and CHIKV, DNA fragments were obtained by PCR using infectious clones (for JEV III, a mutation was corrected using fusion PCR as described in the Supplementary Methods). For JEV I (all DNA fragments), DENV-4 (first and third fragments) and YFV (first and third fragments), DNA fragments were synthesized *de novo* (Genscript) and amplified by PCR. Amplicons were produced using the Platinum PCR SuperMix High Fidelity kit (Life Technologies). The mixture (final volume, 50 µl) consisted of 45 µl SuperMix, 2 µl DNA template at 1 ng µl^−1^ (infectious clone or synthesized DNA fragment) and 200 nM of each primer. For DENV-4 and YFV, the second DNA fragment was obtained by RT-PCR from clarified cell supernatants. Nucleic acids were extracted using the EZ1 Virus Mini kit v2 on the EZ1 Biorobot (both from Qiagen) according to the manufacturer’s instructions and amplified with the Superscript III One-Step RT-PCR Platinum *Taq* High Fidelity kit (Life Technologies). The mixture (final volume, 50 µl) contained 25 µl Reaction Mix, 2 µl nucleic acid extract, 100 nM of each primer, 1 µl Enzyme Mix and 20 µl nuclease-free water. Assays were performed on a Biometra TProfessional Standard Gradient thermocycler with the following conditions: 94 °C for 2 min followed by 40 cycles of 94 °C for 15 s, 64 °C for 30 s, 68 °C for 5 min and a preliminary step of 50 °C for 30 min for the RT-PCR. Size of the PCR products was verified by gel electrophoresis and purified using an Amicon Ultra 0.5 ml kit (Millipore) according to the manufacturer’s instructions.

When plasmid DNA was used as template, the complete removal of the template was ensured by a digestion step with the restriction enzyme *Dpn*I (New England Biolabs) before transfection. To control the efficiency of this additional step, we transfected (see below), as a control, only two cDNA fragments (the first and the second, 1 µg final). These controls did not produce any infectious virus.

#### Cell transfection.

A final amount of either 1 µg of an equimolar mix of all cDNA fragments amplified by PCR or 1 µg of infectious clone of CV-B3 was incubated with 12 µl of Lipofectamine 2000 (Life Technologies) in 600 µl of Opti-MEM medium (Life Technologies). According to the manufacturer’s instructions, the mixture was added to a 12.5 cm^2^ culture flask of subconfluent cells containing 1 ml of medium without antibiotics. After 4 h of incubation, the cell supernatant was removed, cells were washed twice in Hank’s balanced salt solution (HBSS; Life Technologies) and 3 ml of fresh medium was added. The cell supernatant was harvested when gross CPE was observed (3–9 days depending on the cell type and the virus growth speed) or 9 days post-transfection for non-cytopathic viruses, clarified by centrifugation, aliquoted and stored at −80 °C. Each virus was then passaged four times using the same cell type except for the DENV-4 and YFV for which VeroE6 and HEK-293 were respectively used. Passages were performed by inoculating 333 µl of clarified cell supernatant onto cells in a 12.5 cm^2^ culture flask containing 666 µl of medium: after 2 h of incubation, cells were washed twice in HBSS and 3 ml of fresh medium was added. The cell supernatant was harvested after 2–6 days, clarified by centrifugation, aliquoted and stored at −80 °C. Clarified cell supernatants (virus stocks) were used to perform quantification of viral RNA, TCID_50_ assay, direct immunofluorescence assay and whole-genome sequencing.

#### Real-time PCR and RT-PCR assays.

To assess the production of infectious viruses and ensure that positive detection was not the result of cDNA contamination, viral RNA was quantified and compared with the quantity of detected cDNA using the Access RT-PCR Core Reagent kit (Promega) with or without the reverse transcriptase. RNA was extracted using the EZ1 mini virus 2.0 kit and the EZ1 Biorobot (both from Qiagen) according to the manufacturer’s instructions.

The mixture (final volume: 25 µl) contained a standard quantity of AMV/Tfl 5X Reaction Buffer, 0.5 µM of each primer, 0.5 µl dNTP Mix, 0.5 mM MgSO_4_, 0.5 µl AMV reverse transcriptase (only for RT-PCR), 0.5 µl Tfl DNA polymerase, 15.5 µl nuclease-free water and 2 µl of extracted nucleic acids. Assays were performed using the CFX96 Touch real-time PCR Detection System (Bio-Rad) with the following conditions: 50 °C for 15 min, 95 °C for 2 min, followed by 45 cycles of 95 °C for 15 s, 60 °C for 40 s (Table S2). Data collection occurred during the 60 °C step. The difference between cycle threshold values (*C*_t_) obtained by RT-PCR and real-time RT-PCR assays was used to assess viral RNA production. In addition, the amount of viral RNA expressed as dose detection limit (arbitrary unit; AU) was calculated from standard curves (nucleic acids from cell supernatants of cultured viruses were used as standard; five nucleic acid extracts were pooled and 10 µl aliquots were stored at −80 °C).

#### TCID_50_ assay.

For each determination, a 96-well plate culture containing 20 000 BHK-21 cells in 100 µl of medium per well (added just before the inoculation) was inoculated with 50 µl of serial 10-fold dilutions of clarified cell culture supernatants. Each row included six wells of the same dilution and two negative controls. The plates were incubated for 7 days and read for absence or presence of CPE in each well. The determination of the TCID_50_ ml^−1^ was performed using the method of Reed & Muench (1938).

#### Direct immuno-fluorescence assay (dIFA).

Direct IFA were performed using 12.5 cm^2^ culture flasks of SW13 cells for JEV I and JEV III, and VeroE6 cells infected respectively 2 and 6 days before using clarified cell supernatant (see passage of viruses, above). The supernatant was removed and the cells washed twice in HBSS, trypsinized, harvested and diluted (1 : 5) with fresh medium. After cytocentrifugation of 150 µl of this cell suspension (3 min, 900 r.p.m.; Cytospin, Thermo Scientific), the slides were dried, plunged 20 min in cold acetone for fixation, dried, incubated 30 min at 37 °C with appropriately diluted JEV-specific immune serum (see above) or monoclonal DENV-specific antibodies, washed twice with PBS, washed once with distilled water, dried, incubated 30 min at 37 °C with the appropriately diluted FITC-conjugated secondary antibody and Evans blue counterstain, washed twice with PBS, washed once with distilled water, dried, mounted and read using a fluorescence microscope.

#### Sequence analysis of the full-length genome.

Complete genome sequencing was performed using the Ion PGM Sequencer (Rothberg *et al.*, 2011) (Life Technologies) and analyses conducted with the CLC Genomics Workbench 6 software. Virus supernatants were first clarified and treated with the Benzonase nuclease HC >99 % (Novagen) at 37 °C overnight. Following RNA extraction (no RNA carrier was used, see above) using the EZ1 mini virus 2.0 kit and the EZ1 Biorobot (both from Qiagen), random amplification of nucleic acids was performed as previously described (Stang *et al.*, 2005). Amplified DNA was analysed using the Ion PGM Sequencer according to the manufacturer's instructions. The read sequences obtained were trimmed, first using quality score, then by removing the primers used during the random amplification and finally at the 5′ and 3′ termini by systematically removing 6 nt. Only reads with a length greater than 29 nt were used and mapped to the original genome sequence used as a reference. Mutation frequencies (proportion of viral genomes with the mutation) for each position were calculated simply as the number of reads with a mutation compared to the reference divided by the total number of reads at that site.
